# Efficacy of Treatment of Non-hereditary Angioedema

**DOI:** 10.1007/s12016-016-8585-0

**Published:** 2016-09-27

**Authors:** Mignon van den Elzen, M. F. C. L. Go, A. C. Knulst, M. A. Blankestijn, H. van Os-Medendorp, H. G. Otten

**Affiliations:** 1 Division of Internal Medicine and Dermatology, University Medical Center Utrecht, Utrecht, The Netherlands; 2 Department of Dermatology and Allergology, University Medical Center Utrecht (G02.124), PO Box 85.500, 3508 GA Utrecht, The Netherlands; 3 Laboratory of Translational Immunology, University Medical Center Utrecht, Utrecht, The Netherlands

**Keywords:** Angioedema, Angiotensin-converting enzyme inhibitor, Idiopathic, Treatment, Wheals

## Abstract

Non-hereditary angioedema (AE) with normal C1 esterase inhibitor (C1INH) can be presumably bradykinin- or mast cell-mediated, or of unknown cause. In this systematic review, we searched PubMed, EMBASE, and Scopus to provide an overview of the efficacy of different treatment options for the abovementioned subtypes of refractory non-hereditary AE with or without wheals and with normal C1INH. After study selection and risk of bias assessment, 61 articles were included for data extraction and analysis. Therapies were described for angiotensin-converting enzyme inhibitor-induced AE (ACEi-AE), for idiopathic AE, and for AE with wheals. Described treatments consisted of ecallantide, icatibant, C1INH, fresh frozen plasma (FFP), tranexamic acid (TA), and omalizumab. Additionally, individual studies for anti-vitamin K, progestin, and methotrexate were found. Safety information was available in 26 articles. Most therapies were used off-label and in few patients. There is a need for additional studies with a high level of evidence. In conclusion, in acute attacks of ACEi-AE and idiopathic AE, treatment with icatibant, C1INH, TA, and FFP often leads to symptom relief within 2 h, with limited side effects. For prophylactic treatment of idiopathic AE and AE with wheals, omalizumab, TA, and C1INH were effective and safe in the majority of patients.

## Introduction

Angioedema (AE) frequently occurs as part of urticaria, a disease characterized by the development of wheals, AE, or both [1, 2]. AE with wheals, also known as chronic spontaneous urticaria (CSU), is presumably mast cell-mediated [1–3]. AE without significant wheals can be the presenting symptom of a variety of diagnoses, such as hereditary AE caused by C1 esterase inhibitor (C1INH) deficiency, resulting in the release of the key mediator bradykinin [2]. Accumulation of bradykinin can also be caused by the use of angiotensin-converting enzyme inhibitors (ACEi-AE) in patients with normal C1INH [2, 4]. ACEi-AE is estimated to occur in up to 0.68 % of patients who receive ACE inhibitors [5]. However, a majority of patients suffer idiopathic acquired AE, which implies AE with normal C1INH with no family history of AE, in which known causes of AE have been excluded [2, 3]. It is unclear to what extent idiopathic AE is similar to angioedema with wheals (CSU) [3], or to presumably bradykinin-mediated subtypes of AE.

Second-generation antihistamines are used as prophylactic treatment of AE with wheals and idiopathic AE [1, 2]. Antihistamines and corticosteroids, and, in life-threatening cases, adrenaline, represent the standard emergency room treatment of acute attacks of AE [2, 4, 6, 7]. CSU is thought to affect 0.5–1 % of the global population at any given time, with an estimated 67 % of patients with CSU shown to have both hives and AE and 1–13 % to have AE alone [8, 9]. In AE with wheals, daily treatment with antihistamines does not always lead to a complete absence of symptoms [1], and it is estimated that every third or fourth patient remains symptomatic even despite high-dose antihistamine treatment [8, 9]. Omalizumab is effective in patients with CSU [1, 10–15], although it has not been studied extensively in AE without wheals. Patients with ACEi-AE generally do not respond to conventional therapy [5, 6]. Pathophysiology suggests that drugs registered for hereditary angioedema (HAE) due to C1INH deficiency could also be effective in ACEi-AE. Several drugs are currently available, including (1) antifibrinolytic agents such as tranexamic acid (TA); (2) attenuated androgens such as danazol; (3) replacement of deficient proteins using fresh frozen plasma (FFP); (4) C1INH concentrates, which inhibit the formation of bradykinin; (5) the selective plasma kallikrein inhibitor ecallantide; and (6) the selective bradykinin B2 receptor antagonist icatibant [2]. Some of these drugs are licensed to treat acute attacks, whereas others are used for prophylactic treatment [2]. The efficacy of these drugs in refractory AE with normal C1INH has not been fully elucidated.

This systematic literature review aims to provide an overview of therapeutic options and their efficacy in patients with AE with normal C1INH, but refractory to conventional therapy. We have distinguished between treatment of acute attacks vs. prophylactic treatment and included bradykinin-mediated and mast cell-mediated non-hereditary AE as well as idiopathic AE.

## Methods

This systematic literature review was conducted using the criteria mentioned in the Preferred Reporting Items of Systematic Reviews and Meta-Analyses (PRISMA) statement [16].

### Search Strategies

Secondary evidence databases National Guideline Clearinghouse, CBO guidelines, Trip Database, and the Cochrane Library were searched for guidelines up to 20th April 2015 using several synonyms for the domain, angioedema, and determinant, treatment options (Table 1). Subsequently, primary evidence electronic databases PubMed, EMBASE, and Scopus were searched for articles up to 20th April 2015 using the domain and determinants as previously described. Synonyms for outcome measurements were not included in the search strategy so as to maximize the yield of articles and to allow for different outcome measures, including but not limited to time to initial or complete response and decrease in attack frequency or severity. The search was limited by title or abstract and, in Scopus, by title, abstract, or keywords.

**Table 1:** Search syntax performed on 20th April 2015 in PubMed, EMBASE, and Scopus

Search
(Angioedema OR ‘angio edema’ OR angioedemas) AND (treatment OR therapy OR antihistamines OR (ciclosporine OR CsA OR cyclosporine) OR (omalizumab OR (anti IgE)) OR (danazol OR ‘attenuated androgen’ OR androgen) OR C1 inhibitor concentrate OR (tranexamic acid OR TTA OR cyklokapron OR AMCA OR ‘trans aminomethyl cyclohexane carboxylic acid’) OR biological OR antileukotrienes OR (‘H2 antagonist’ OR ‘histamine antagonist’) OR (TCA OR antidepressant) OR (icatibant OR ‘bradykinin receptor antagonist’) OR (MTX OR methotrexate) OR (AZA OR azathioprine OR Imuran) OR (corticosteroids OR prednisone OR glucocorticosteroids) OR Adrenaline OR sulphasalazine OR (dapson OR dapsone) OR hydroxychloroquine OR Plasmapheresis OR (‘intravenous immunoglobulin’ OR IVIG)) OR (‘Fresh Frozen Plasma’ OR FFP))

Search term ‘biological’ was entered as ‘biologicals’ in EMBASE database.

### Inclusion and Exclusion Criteria

Articles were included when the described study populations suffered ACEi-AE, AE with wheals (CSU), or idiopathic AE with normal C1INH. Furthermore, only articles describing pharmacological treatment of AE were included. This included both observational studies (case report and case series) and intervention trials (cohort studies or randomized controlled trials, RCTs). Any pharmacological treatment other than antihistamines up to fourfold, prednisolone, or adrenaline could be included. Articles were considered appropriate to be included only when sufficient details regarding type of treatment, dose, interval between doses, time to initial response, and time to maximum or complete response were described. Articles describing only ineffective therapies were described separately. For full understanding of scientific content by the authors who performed the selection of studies, only articles written in English, Dutch, or German were included. Recent articles for which only the title and abstract were available, such as congress abstracts, were included only if sufficient information about the patient(s), treatment regimen, and response were described. For icatibant only, when dose was missing, it was assumed that 30 mg was used due to the packaging of this product. Therefore, these articles could be included, although the dose was shown as “not reported” in the results. Articles regarding AE with wheals could only be included when treatment results specifically for AE symptoms could be extracted rather than only for the symptoms wheals and itch. Outcome measurements could differ for articles regarding an acute attack or prophylactic treatment: in acute settings, initial and complete responses refer to the resolution of a single attack of swelling, whereas in prophylactic or chronic settings this refers to a decrease in attack frequency or severity.

Studies were excluded when AE was caused by hereditary or acquired complement C1 inhibitor (C1-INH) deficiency, coagulation factor 12 (fXII) mutation, formerly known as HAE type 3, or other known causes of AE, including allergy, or when AE was an adverse effect of any therapy other than ACEi.

### Selection of Studies

Unique titles and abstracts and subsequently full texts were screened for eligibility. Articles published in or after 2013 were screened by at least two independent reviewers (ME, MG, and MB), results were compared, and disagreements were discussed and resolved. Articles published before 2013 were screened by one reviewer (ME), and for assessment of unclear articles only, a second reviewer (MB) was available.

### Risk of Bias

Risk of bias (RoB) for each study was assessed by one reviewer (MG) and verified by a second reviewer (ME). To allow for a careful assessment of observational studies as well as intervention studies, criteria for risk of bias assessment from the Cochrane Handbook for Systematic Reviews of Interventions [17] were supplemented with items from the CARE guidelines checklist [18]. The risk of selection bias, performance bias, detection bias, attrition bias, and selective reporting bias was assessed. A low risk of bias was preferred and therefore displayed as a positive finding (+), whereas a high risk of bias was undesirable and displayed as a negative finding (−). The risk of selection bias was considered low (+) for observational studies when symptoms and important clinical findings were described. The risk of performance bias was considered low (+) when the chosen treatment option and dose regimen were both recorded. The risk of detection bias was assessed with regard to (1) effect of treatment and (2) adverse events and was considered low if this was noted in the article. In case of multiple patient groups including more than one type of AE, the risk of detection bias was considered low only when results could be extracted for the subgroups separately and unclear (+/−) when results were described for the total group. The risk of attrition bias was low (+) when reasons for exclusion or dropout were reported, and for controlled studies, the dropouts were balanced between treatment and placebo groups. The risk for reporting bias was low (+) when all prespecified outcomes were fully addressed in the results. Authors of RCTs were contacted to retrieve missing trial details. All evaluations were compared and disagreements between authors were discussed and resolved.

### Data Extraction and Synthesis

For each study, data extraction was performed by one reviewer and verified by a second reviewer (ME and MG). Data regarding the study design, therapy, previous therapies, and effect of the described therapy were recorded in tables. For treatment of acute attacks and prophylactic treatment of AE, available efficacy results were described per subtype and per treatment option. Definitions for response were adopted from the original articles. Articles describing ineffective treatment options were described separately. If information about adverse effects was available, this was collected additionally for each type of treatment. A distinction between serious adverse effects (SAEs) and less severe treatment-emergent adverse events (TEAEs) was made. Additionally, only adverse events possibly, probably, or definitely related to treatment were reported. Adverse effects reported by placebo-treated patients were not taken into account. Due to the high amount of available case reports and low amount of controlled studies, and since outcome measures varied among the study studies, a meta-analysis could not be performed. Instead, results are described using narrative summary technique.

## Results

### Search Results and Quality Assessment

**Fig. 1:**
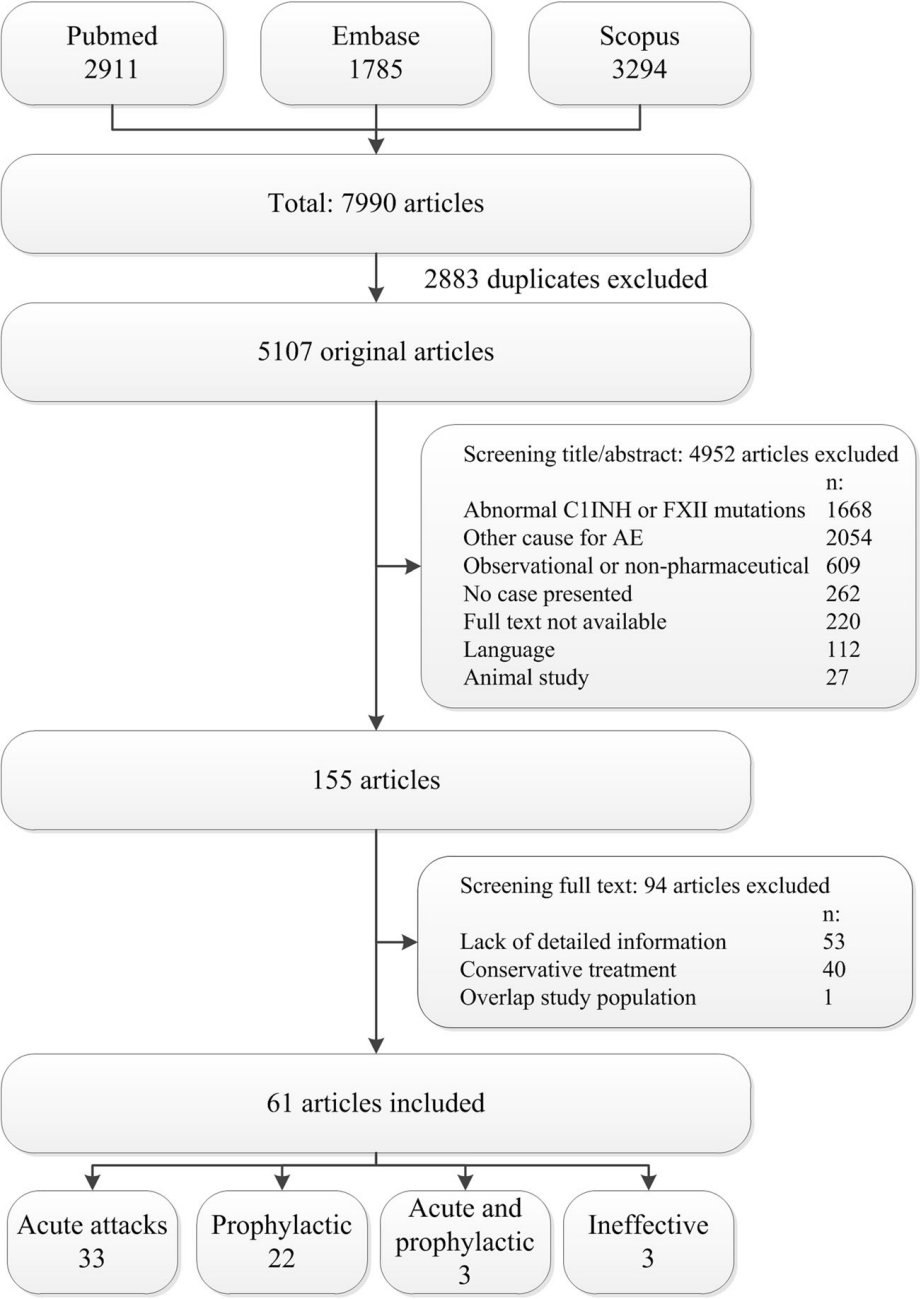
Flowchart of the included and excluded articles

The search in secondary evidence databases yielded no available aggregated evidence. The search in PubMed, EMBASE, and Scopus yielded 5107 original articles (Fig. 1). After screening titles and abstracts, 4952 articles were excluded. Subsequently, 155 full texts were screened for eligibility, leading to the exclusion of 94 further articles, including 53 articles with a lack of usable information, the use of conservative treatment in 40 articles, and overlap in study population in one article. The remaining 61 articles included 53 full articles and eight (congress) abstracts. Of the 61 included articles, 38 described treatment of AE in acute settings, including 3 RCTs, 2 cohort studies, 4 case series, and 29 case reports. Additionally, 26 of the 61 articles described prophylactic settings, including 1 RCT, 5 cohort studies, 9 case series, and 11 case reports. Three articles described both acute and prophylactic treatment.

All 61 articles underwent RoB assessment (Tables 2 and 3). All of the four included RCTs had a low risk of bias. Of the 57 descriptive studies, 46 had a low risk of bias and 11 had an unclear risk of at least one type of bias. Only 26 addressed safety results with regard to efficacy outcomes.

**Table 2:** Risk of bias of acute setting studies

Acute setting				Selection bias			Performance bias		Detection bias			Attrition bias	Reporting bias	Remarks
Study	Design	AE subtype	Usable sample size	Randomization	Allocation concealment	Case description	Blinding patient and personnel	Intervention	Blinding outcome	Assessment outcome	Adverse events	Incomplete outcome data	Selective reporting	
Lewis [4]	RCT	ACEi-induced	58 + 18	+	+	na	+	na	+	na	na	+	+	
Bas [5]	RCT	ACEi-induced	13 + 14	+	+	na	+	na	+	na	na	+	+	
Bernstein [7]	RCT	ACEi-induced	26 + 24	+	+	na	+	na	+	na	na	+	+	
Mansi [19]	Cohort	Idiopathic	26	na	na	+	na	+	na	+/−	−	+	+	^a^
Bouillet [20]	Cohort	Idiopathic	48	na	na	+/-	na	+/−	na	+/−	−	+	+	
Bova [21]	CS	ACEi-induced	13	na	na	+	na	+	na	+	+	na	na	
Greve [22]	CS	ACEi-induced	10	na	na	+	na	+	na	+	+	na	na	
Bas [6]	CS	ACEi-induced	8	na	na	+	na	+	na	+	+	na	na	
Hassen [23]	CS	ACEi-induced	7	na	na	+	na	+	na	+	−	na	na	
Bartal [24]	CR	ACEi-induced	1	na	na	+	na	+	na	+	−	na	na	
Lipski [25]	CR	ACEi-induced	1	na	na	+	na	+	na	+	−	na	na	
Charmillon [26]	CR	ACEi-induced	1	na	na	+	na	+	na	+	−	na	na	
Crooks [27]	CR	ACEi-induced	1	na	na	+	na	+	na	+	+	na	na	
Rasmussen [28]	CR	ACEi-induced	1	na	na	+	na	+	na	+	−	na	na	
Yates [29]	CR	ACEi-induced	1	na	na	+	na	+	na	+	−	na	na	
Bledsoe [30]	CR	ACEi-induced	1	na	na	+	na	+	na	+	−	na	na	
Volans [31]	CR	ACEi-induced	2	na	na	+	na	+	na	+	−	na	na	
Bolton [32]	CR	ACEi-induced	1	na	na	+	na	+	na	+	−	na	na	
Gallitelli [33]	CR	ACEi-induced	1	na	na	+	na	+	na	+	−	na	na	
Millot [34]	CR	ACEi-induced	1	na	na	+	na	+	na	+	−	na	na	
Stewart [35]	CR	ACEi-induced	2	na	na	+	na	+	na	+	−	na	na	
Bas [36]	CR	ACEi-induced	1	na	na	+	na	+	na	+	−	na	na	
Schmidt [37]	CR	ACEi-induced	1	na	na	+	na	+	na	+	−	na	na	
Dehne [38]	CR	ACEi-induced	1	na	na	+	na	+	na	+	−	na	na	
Nielsen [39]	CR	ACEi-induced	1	na	na	+	na	+	na	+	−	na	na	
Karim [40]	CR	ACEi-induced	1	na	na	+	na	+	na	+	−	na	na	
Bertazzoni [41]	CR	Idiopathic	1	na	na	+	na	+	na	+	−	na	na	
Nanda [42]	CR	Idiopathic	1	na	na	+	na	+	na	+	+	na	na	
Stahl [43]	CR	Idiopathic	1	na	na	+	na	+	na	+	−	na	na	^a^
Montinaro [44]	CR	Idiopathic	1	na	na	+	na	+	na	+	−	na	na	
O’Keefe [45]	CR	Idiopathic	1	na	na	+	na	+	na	+	−	na	na	
Lleonart [46]	CR	Idiopathic	1	na	na	+	na	+	na	+	+	na	na	
Sridhara [47]	CR	Idiopathic	1	na	na	+	na	+	na	+	−	na	na	
Vela Vizcaino [48]	CR	Idiopathic	1	na	na	+	na	+	na	+/−	−	na	na	^a^
Colas [49]	CR	Idiopathic	1	na	na	+	na	+	na	−	−	na	na	
Seoane [50]	CR	Idiopathic	1	na	na	+/−	na	+/-	na	−	−	na	na	
Illing [51]	CR	ACEi-induced	1	na	na	+	na	+	na	+	−	na	na	Ineff.
Tran [52]	CR	Idiopathic	1	na	na	+/−	na	+	na	+	+/−	na	na	Ineff.

+: low risk of bias, − high risk of bias, +/− unclear risk of bias

*AE* angioedema, *RCT* randomized controlled trial, *CS* case series, *CR* case report, *ACEi* angiotensin-converting enzyme inhibitor, *na* not applicable, *Ineff* ineffective treatment described in the specific article

^a^See also prophylactic setting table

**Table 3:** Risk of bias of prophylactic setting studies

Prophylactic setting				Selection bias			Performance bias		Detection bias			Attrition bias	Reporting bias	Remarks
Study	Design	AE subtype	Usable sample size	Randomization	Allocation concealment	Case description	Blinding patient and personnel	Intervention	Blinding outcome	Assessment outcome	Adverse events	Incomplete outcome data	Selective reporting	
Zazzali [53]	RCT	AE with wheals	208	+^a^	+^a^	na	+^a^	na	+^a^	na	na	+	+	^a^
Rijo Calderón [54]	Cohort	AE with wheals	10	na	na	+/−	na	+/−	na	+/−	+	na	na	
	Cohort	Idiopathic	4	na	na	+/−	na	+/−	na	+/−	+	na	na	
Mansi [19]	Cohort	Idiopathic	44	na	na	+	na	+	na	+	+	+	+	^b^
Wintenberger [55]	Cohort	Idiopathic	25	na	na	+	na	+	na	+	+	+	+	
Firinu [56]	Cohort	Idiopathic	16	na	na	+	na	+	na	+	+/−	+	+	
Saule [57]	Cohort	Idiopathic	20	na	na	+	na	+/−	na	+	+	na	na	
Du-Thanh [58]	CS	Idiopathic	25	na	na	+	na	+	na	+	+	na	na	
Cicardi [59]	CS	Idiopathic	15	na	na	+	na	+	na	+	+	na	na	
Azofra [60]	CS	Idiopathic	8	na	na	+	na	+	na	+	+	na	na	
Sands [61]	CS	Idiopathic	3	na	na	+	na	+	na	+	−	na	na	
vd Elzen [62]	CS	AE with wheals	3	na	na	+	na	+	na	+	+/−	na	na	
Groffik [63]	CS	AE with wheals	2	na	na	+	na	+	na	+	+/−	na	na	
Büyüköztürk [64]	CS	AE with wheals	1	na	na	+	na	+	na	+	+	na	na	
	CS	Idiopathic	2	na	na	+	na	+	na	+	+	na	na	
Perez [65]	CS	Idiopathic	2	na	na	+	na	+	na	+	+	na	na	
Ghazanfar [66]	CR	AE with wheals	1	na	na	+	na	+	na	+	+	na	na	
Wieder [67]	CR	AE with wheals	1	na	na	+	na	+	na	+	−	na	na	
Kutlu [68]	CR	AE with wheals	1	na	na	+	na	+	na	+	−	na	na	
Ozturk [69]	CR	AE with wheals	1	na	na	+	na	+	na	+	−	na	na	
Sánchez-Machín [70]	CR	AE with wheals	1	na	na	+	na	+	na	+	+	na	na	
Korkmaz [71]	CR	AE with wheals	1	na	na	+	na	+	na	+	−	na	na	
Stahl [43]	CR	Idiopathic	1	na	na	+	na	+	na	+	−	na	na	^b^
von Websky [72]	CR	Idiopathic	1	na	na	+	na	+	na	+	+	na	na	
Suna [73]	CR	Idiopathic	1	na	na	+	na	+	na	+	+	na	na	
Bayer [74]	CR	Idiopathic	1	na	na	+	na	+/−	na	−	−	na	na	
Vela Vizcaino [48]	CR	Idiopathic	1	na	na	+	na	+	na	+/−	−	na	na	^b^
Maggadottir [75]	CS	AE with wheals	2	na	na	+/−	na	+/−	na	+/−	−	na	na	Ineff.

+: low risk of bias, − high risk of bias, +/− unclear risk of bias

*AE* angioedema, *RCT* randomized controlled trial, *CS* case series, *CR* case report, *ACEi* angiotensin-converting enzyme inhibitor, *na* not applicable, *Ineff*. ineffective treatment described in the specific article

^a^Study procedures described in separate articles

^b^See also prophylactic setting table

### Treatment of Acute Attacks of AE

With regard to acute attacks of AE refractory to conventional treatment including antihistamines, corticosteroids, and adrenaline, the included articles described treatment of two subtypes: ACEi-AE and idiopathic AE.

**Fig. 2:**
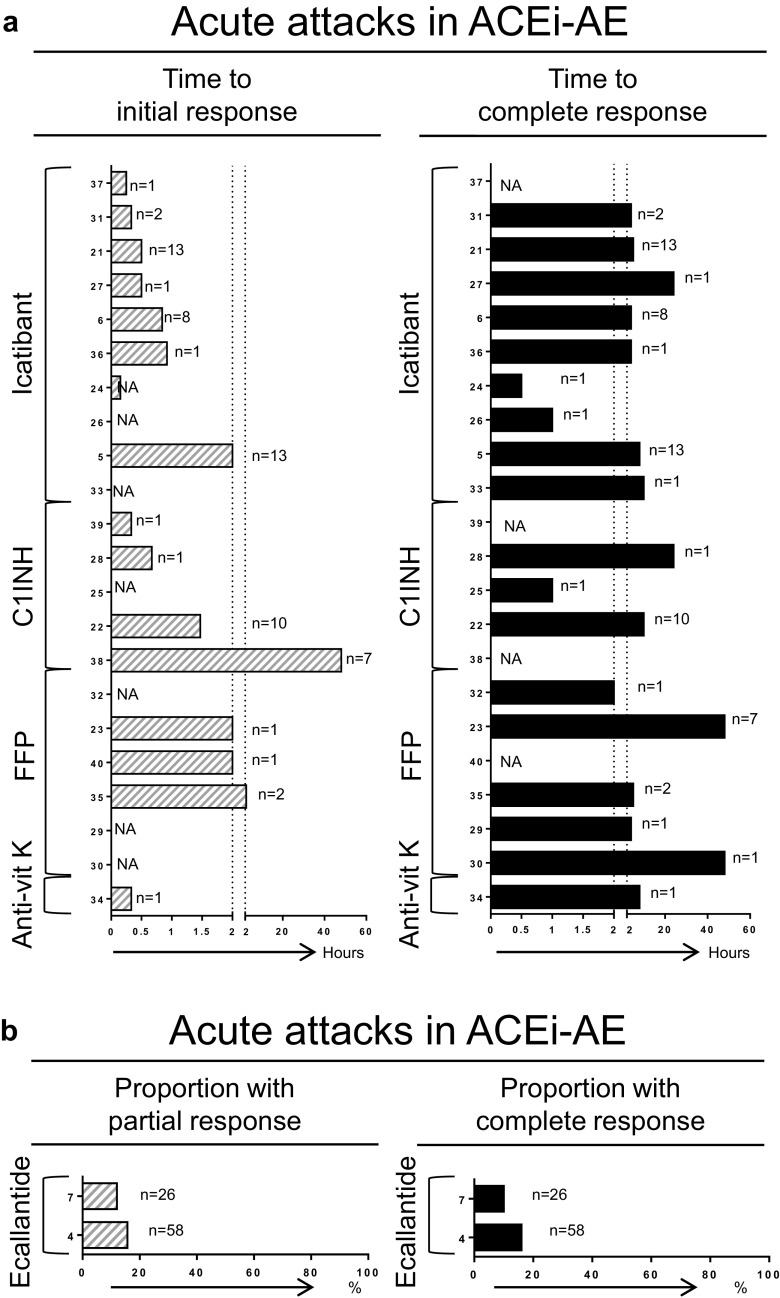

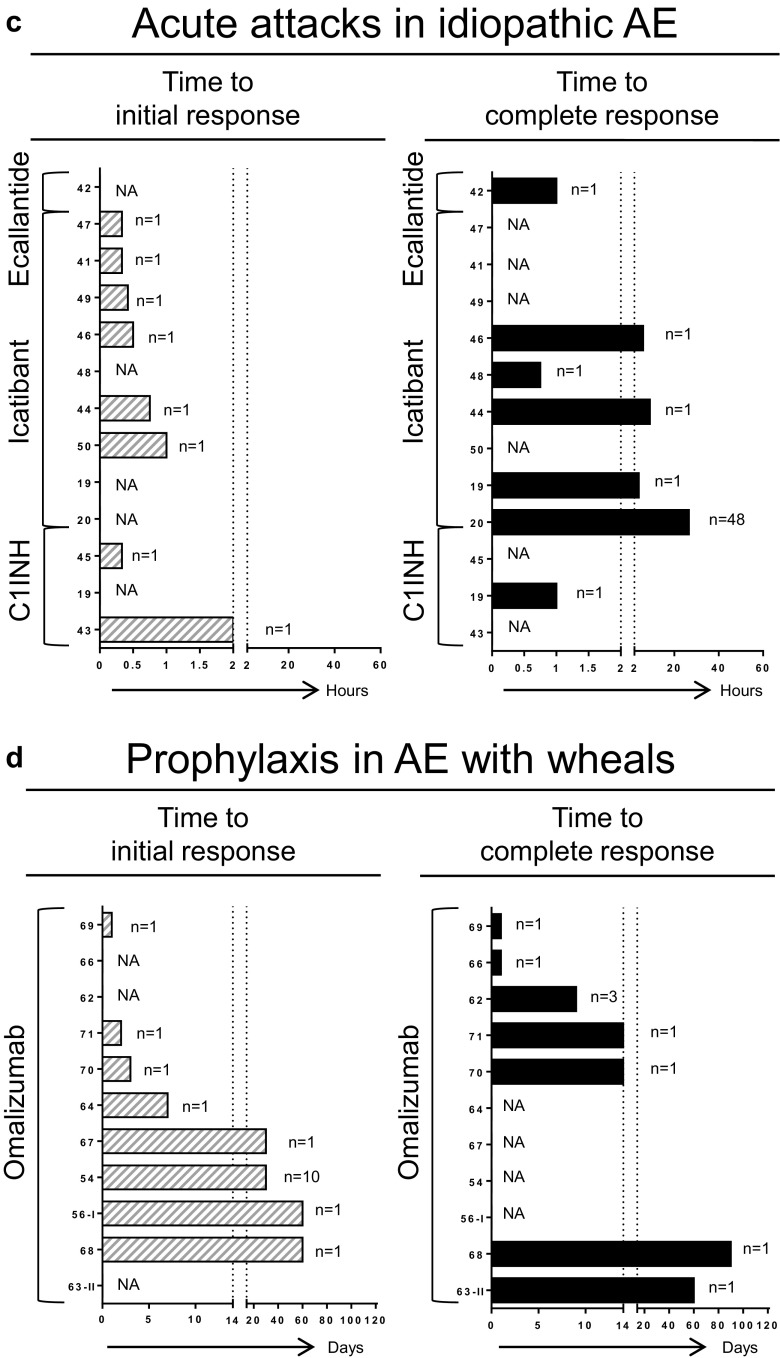

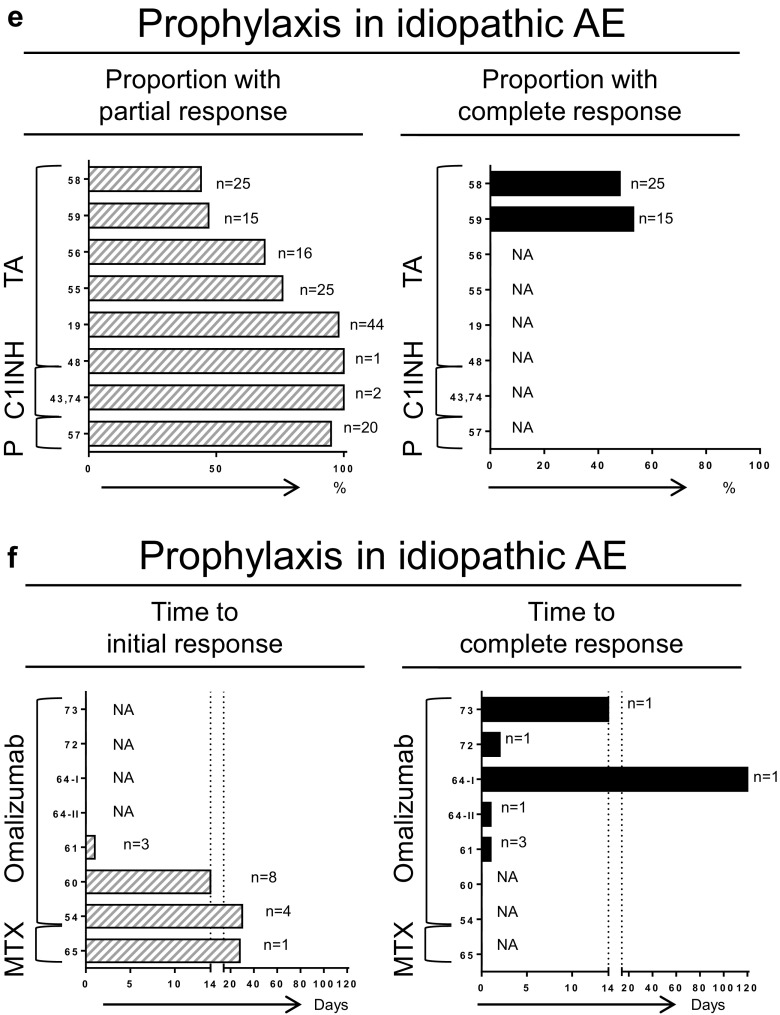
**a**–**d** Responses to treatment. *NA* not available, *Anti-vit K* anti-vitamin K, *C1INH* complement 1 esterase inhibitor, *MTX* methotrexate, *TA* tranexamic acid, *P* progestin. *Numbers on the Y-axis* represent the reference number for each study; *n* indicates the number of patients included from each study. Not shown in (**c**): Mansi et al., 13 of 24 patients had partial response to tranexamic acid. Not shown in (**d**): Zazzali et al., in 208 patients treated with omalizumab, the mean proportion of AE-free days was 90.1–95.8 % vs. 88.7 % for placebo

ACEi-AE was addressed in 24 articles describing treatment of acute attacks in 154 patients, with study sizes varying from 1 to 58 patients. Outcome measures were (1) time to response (Fig. 2a and Table 4) and/or (2) proportion of patients with response (Fig. 2b and Table 4). As shown in Fig. 2a, described treatment strategies consisted of icatibant (42 patients in ten articles including one RCT) [5, 6, 21, 24, 26, 27, 31, 33, 36, 37], C1INH (14 patients in five articles) [22, 25, 28, 38, 39], FFP (13 patients in six articles) [23, 29, 30, 32, 35, 40], and kanokad (concentrate of vitamin K-dependent coagulation factor anti-vitamin K antagonist in one patient using anti-vitamin K medication concomitantly) [34]. In the 21 included studies for icatibant, C1INH, and FFP, the (median) time to initial response ranged from a few minutes up to 150 min, with one outlier up to 48 h [38]. Time to complete response ranged from 0.5 to 48 h. As shown in Fig. 2b, ecallantide was described additionally in 84 patients in two RCTs [4, 7]. Results for ecallantide were not significant: one RCT identified a difference in response rate vs. placebo of 16 % (95 % confidence interval, −11 to 41 %) [4], and a second RCT revealed a difference in response rate vs. placebo of 10 % (95 % confidence interval, −14 to 34 %) [7]. The level of evidence for C1INH, FFP, and icatibant was low, compared to ecallantide, due to lack of controlled studies. In conclusion, in treatment of acute attacks of ACEi-AE, no significant differences in the response rate between ecallantide and placebo were shown, and icatibant, C1INH, and FFP had similar times to response, mostly less than 2 h.

**Table 4:** Results of acute setting studies: subtype ACEi-induced angioedema

Author	Year	Study design	Size	Previous therapy	Therapy	Dosage	Effect
Lewis [4]	2015	RCT	58 + 18	AH + C + E + H2	Ecallantide	10–60 mg	Predefined criteria ≤6 h met in 88 vs. 72 % for PLC (difference = 16 %; 95%CI = 11–41)
Bernstein [7]	2015	RCT	26 + 24	AH + C + E	Ecallantide	30 mg	Discharge criteria ≤4 h in 31 vs. 21 % for PLC (difference = 10 %; 95 %CI = 14–34 %)
Bas [5]	2015	RCT	13 + 14	None	Icatibant	30 mg	Median IR = 120 min (95%CI = 60–480) vs. 702 min (480–1080); CR = 8.0 vs. 27.1 h (3.0–16.0)
Bova [21]	2015	CS	13	AH + C + E	Icatibant	30 mg	IR = 30 min (IQR = 27.5–70); CR = 5 h (IQR = 4–7)
Bas [6]	2010	CS	8	None	Icatibant	30 mg	IR = 50.6 min (SD = 21); CR = 4.4 h (SD = 0.8)
Volans [31]	2013	CS	2	AH + C + E + TA	Icatibant	30 mg	IR = 20 min; CR = 4 h
Bartal [24]	2015	CR	1	AH + C + E + H2	Icatibant	30 mg	IR within minutes; CR = 0.5 h
Charmillon [26]	2014	CR	1	n.r.	Icatibant	30 mg	CR = 1 h
Crooks [27]	2014	CR	1	AH + C + E	Icatibant	30 mg	IR = 30 min; CR = 24 h
Gallitelli [33]	2012	CR	1	None	Icatibant	30 mg	CR = 10 h
Bas [36]	2011	CR	1	C	Icatibant	30 mg	IR = 55 min; CR = 4 h
Schmidt [37]	2010	CR	1	AH + C + E + C1INH	Icatibant	30 mg	IR = 15 min
Greve [22]	2014	CS	10	None	C1INH (B)	1000 U	IR = 88 min (SD = 38); CR = 10.1 h (SD = 3)
Lipski [25]	2015	CR	1	C + E + FFP	C1INH (B)	20 U/kg	CR < 1 h
Rasmussen [28]	2014	CR	1	None	C1INH	15 U/kg	IR = 40 min; CR < 24 h
Dehne [38]	2007	CR	1	AH + C + E + P + FFP	C1INH (B)	1000 IE	IR = 2 days after initial worsening in the first 24 h
Nielsen [39]	2006	CR	1	AH + C	C1INH (B)	1500 U	IR = 20 min
Hassen [23]	2013	CS	7	AH + C + E + H2	FFP	1-3 U	IR = 2 h; CR = 48 h
Stewart [35]	2012	CR	2	C	FFP	2 U	IR = 2.5 h in 1 patient; CR = 4.75 in the other
Yates [29]	2014	CR	1	None	FFP	2 U	CR = 4 h
Bledsoe [30]	2013	CR	1	AH + C + E + H2	FFP	2 U	IR within a few hours. CR < 48 h
Bolton [32]	2012	CR	1	Not known	FFP	2 U	CR = 2 h
Karim [40]	2002	CR	1	AH + C	FFP	4 U	IR < 2 h
Millot [34]	2012	CR	1	AH + C + E	Kanokad	1500 U	IR = 20 min; CR = 8 h

For*Size*, only the number of patients included for describing therapies are presented in the table. In controlled studies, the number of patients treated with study medication vs. those treated with placebo or comparative treatment are presented as *x* + *y*. The effect of treatment is presented as initial response (*IR*) and complete response (*CR*)

*CS* case series, *CR* case report, *ACEi* angiotensin-converting enzyme inhibitor, *n.r.* not reported, *AH* antihistamine, *C* corticosteroids, *E* epinephrine, *C1INH* C1 inhibitor concentrate (B: Berinert P), *TA* tranexamic acid, *H2* H2 antagonist, *FFP* fresh frozen plasma, *P* pantoprazole, *PLC* placebo

Idiopathic AE was addressed in 12 articles describing treatment of acute attacks in 84 patients. Effect of treatment was described as time to response (Fig. 2c and Table 5) or proportion of patients with response (Table 5). Treatment strategies consisted of icatibant (56 patients in nine studies) [19, 20, 41, 44, 46–50], TA (24 patients in one study) [19], C1INH (three patients in three articles) [19, 43, 45], and ecallantide (one patient) [42]. As shown in Fig. 2c, the time to initial response for C1INH ranged from 20 to 120 min and for icatibant from 20 to 45 min, and (median) time to complete response for ecallantide was 1 h. For C1INH, (median) time to complete response was also 1 h, and for icatibant this ranged from 45 min up to 26 h. In addition to Fig. 2c, one study reported response to TA in 13 of 24 patients (54 %) [19]. In conclusion, in acute attacks of idiopathic AE, C1INH, icatibant, and ecallantide had times to response often within 2 h, and TA was effective in more than 50 % of patients.

**Table 5:** Results of acute setting studies: subtype idiopathic angioedema

Author	Year	Study design	Size	Previous therapy	Therapy	Dosage	Effect
Bouillet [20]	2014	Cohort	48	Unknown	Icatibant	n.r.	Median time to CR = 26.6 h (IQR = 8.3–46)
Bertazzoni [41]	2015	CR	1	AH + C + E	Icatibant	30 mg	IR = 20 min
Seoane [50]	2014	CR	1	AH + C	Icatibant	n.r.	“Rapid response”
Vela Vizcaino [48]	2014	CR	1	AH + C + E	Icatibant	n.r.	CR = 45 min
Montinaro [44]	2013	CR	1	AH + C	Icatibant	30 mg	IR = 45 min; CR = 9 h
Colás [49]	2012	CR	1	AH + C + E + H2 + C1INH	Icatibant	30 mg	IR = 25 min
Lleonart [46]	2012	CR	1	AH + C + E	Icatibant	30 mg	IR = 30 min; CR = 6 h
Sridhara [47]	2012	CR	1	AH + C + E + H2 + H + LTRA	Icatibant	30 mg	IR = 20 min
Mansi [19]	2014	Cohort	1	None	Icatibant	30 mg	CR = 4 h
	2014	Cohort	24	None	TA	≤6 g/day	Decreased severity and duration of symptoms in 13 (54 %)
	2014	Cohort	1	None	C1INH	1000 U	CR = 1 h
Stahl [43]	2014	CR	1	AH + C + E + H2 + TA + H + AB + LTRA + FFP + Icatibant	C1INH	20 U/kg	IR = 2 h
O’Keefe [45]	2013	CR	1	C + E	C1INH	500 U	IR = 20 min
Nanda [42]	2014	CR	1	AH + C + E	Ecallantide	30 mg	CR < 1 h

The effect of treatment is presented as initial response (*IR*) and complete response (*CR*)

*CS* case series, *CR* case report, *n.r.* not reported, *AH* antihistamine, *C* corticosteroids, *E* epinephrine, *C1INH* C1 inhibitor concentrate, *TA* tranexamic acid, *H2* H2 antagonist, *FFP* fresh frozen plasma, *LTRA* leukotriene receptor antagonist, *H* hormones, *AB* antibiotics, *H* hydroxychloroquine

### Prophylactic Treatment of AE

With regard to recurrent AE refractory to conventional treatment, included articles about prophylactic treatment described two subtypes: AE with wheals and idiopathic AE.

AE with wheals was addressed in 11 articles describing 230 patients. Effect was shown as time to response (Fig. 2d and Table 6) [53, 54, 62–64, 66–71]. All articles described treatment with omalizumab after unsuccessful treatment with antihistamines and often additional ineffective treatment options. One manuscript detailed two RCTs for which the results regarding urticaria had been published previously [10, 14]. However, in the included manuscript, specific results with regard to AE were described [53]. In the other articles, which consisted of cohort studies and case series or case reports, the time to initial effect ranged from 1 day to 60 days after administration, and 10 of 22 patients achieved complete remission within a time range varying from 1 day to <150 days [54, 62–64, 66–71]. In conclusion, in prophylactic treatment of AE with wheals, omalizumab had a broad range of time to response and was effective in almost half of the patients.

**Table 6:** Results of prophylactic setting studies: subtype angioedema with wheals

Author	Year	Study design	Size	Disease duration (years)	Previous therapy	Therapy	Dosage scheme	Effect	Follow-up (months)
Zazzali [53]	2014	RCT	208	n.r.	AH	OMA	75–300/4	Mean proportion AE-free days = 90.1–95.8 % vs. 88.7 %	3
Rijo Calderón [54]	2013	Cohort	10	n.r.	AH + C + dapsone	OMA	150–300/2–4	No further attacks in 5, mild symptoms in 7^a^	n.r.
vd Elzen [62]	2014	CS	3	2,4,9	AH + C + LTRA + H2 + AB + I + MTX + HC	OMA	150–300/2–4	CR < 9 days	24
Groffik [63]	2010	CS-1	1	1	AH + C + LTRA	OMA	300/2	IR < 2 months	4
		CS-2	1	19	AH + C + LTRA	OMA	150/2	CR < 2 months	4
Büyüköztürk [64]	2012	CS	1	7	AH	OMA	225/4	IR <1 week	8
Ghazanfar [66]	2015	CR	1	n.r.	AH + C + I	OMA	150/2 → 300/4	CR < 1 day	48
Wieder [67]	2015	CR	1	n.r.	AH + C + LTRA + I + O	OMA	300/4	IR after first dose	29
Kutlu [68]	2014	CR	1	n.r.	AH + C + E	OMA	300/4	IR = 2 months; CR = 3 months	3
Ozturk [69]	2014	CR	1	n.r.	AH + C + O	OMA	300/4	No further attacks	3
Sánchez-Machín [70]	2011	CR	1	9	AH + C + I + O	OMA	300/2 → 300/6	IR = <3 days; CR = 14 days	36
Korkmaz [71]	2010	CR	1	n.r.	AH + C + LTRA + H2 + AB + I	OMA	300/2	IR = 2 days; CR = 14 days	n.r.

Dosage scheme presented as milligrams administered every *x* weeks. An arrow indicates a dose adjustment during the treatment period. The effect of treatment is presented as initial response (*IR*) and complete response (*CR*)

*CS* case series, *CR* case report, *n.r.* not reported, *AH* antihistamine, *C* corticosteroids, *E* epinephrine, *C1-inh* C1 inhibitor concentrate, *TA* tranexamic acid, *H2* H2 antagonist, *LTRA* leukotriene receptor antagonist, *AB* antibiotics, *I* other immunosuppressant, *MTX* methotrexate, *HC* hydroxychloroquine, *O* other therapy

^a^Results mentioned for the whole study population, which may be larger than the patients included in this review

Lastly, prophylactic treatment of idiopathic AE was addressed in 16 articles describing 168 patients [19, 43, 48, 54–61, 64, 65, 72–74]. Efficacy was shown as proportion of patients with response (Fig. 2e and Table 7) or time to response (Fig. 2f and Table 7). As shown in Fig. 2e, described treatment options were TA (126 patients in six studies) [19, 48, 55, 56, 58, 59], progestin (20 patients in one study) [57], and C1INH (two patients in two studies) [43, 74]. When combining studies, TA led to improvement of symptoms in 92 patients (73 %) and a complete absence of symptoms in another 20 patients (16 %; Table 7). Progestin provided improvement in 19 of 20 patients and C1INH in two of two patients. Figure 2f shows the results for omalizumab (19 patients in six articles) [54, 60, 61, 64, 72, 73] and methotrexate (MTX, one patient) [65]. For omalizumab, in 12 patients (63 %), no further attacks occurred after starting treatment, and the time to initial response ranged from 1 day to 120 days. MTX provided improvement in one patient after 28 days of treatment. In conclusion, in prophylactic treatment of idiopathic AE, TA, omalizumab, and C1INH, as well as progestin and MTX, were effective in a majority of patients.

**Table 7:** Results of prophylactic setting studies: subtype idiopathic angioedema

Author	Year	Study design	Size	Disease duration (years)	Previous therapy	Therapy	Dosage scheme	Effect	Follow-up (months)
Mansi [19]	2014	Cohort	44	n.r	n.r.	TA	3 g/day → 0.5–3 mg/day	Reduction recurrences in 43 (98 %)	n.r.
Wintenberger [55]	2014	Cohort	25	n.r.	n.r.	TA	2–2.5 g/day	Attack frequency from 15.2 (range = 2–50) to 3.7 (0–18) per 6 months. No response in 6 (24 %)	6
Firinu [56]	2015	Cohort	16	n.r.	AH + C	TA	1.5–3 g/day	50 % attack frequency decrease in 8 (50 %), no response in 5 (31 %), other in 3	n.r.
Du-Thanh [58]	2010	CS	25	n.r.	AH + C	TA	3 g/day	CR in 12 (48 %), PR in 11 (44 %), no response in 2 (8 %)	20
Cicardi [59]	1999	CS	15	Median 6	AH	TA	3 g/day	No further attacks in 8 (53 %), 7 attack frequency decreased by ≥75 %	10–282
Vela Vizcaino [48]	2014	CR	1	3	AH + C + E + C1INH	TA	3 g/day	Attack frequency decrease from weekly to 3/8 weeks	n.r.
Saule [57]	2012	Cohort	20	n.r.	AH	Progestin	n.r.	Improvement in 19 (95 %)	32,4
Rijo Calderón [54]	2013	Cohort	4	n.r.	AH + C + dapsone	OMA	150–300/2–4	IR < 1 month	n.r.
Azofra [60]	2015	CS	8	n.r.	(AH + C +) TA	OMA	300/4	IR = 2–14 days	6–12 m
Sands [61]	2007	CS-1	1	6	AH + C + E + H2	OMA	300/3	No further attacks	24
		CS-2	1	4	AH + C + H2	OMA	375/2	No further attacks	7
		CS-3	1	9	AH + C + LTRA + H2	OMA	300/4	1 minor attack in 2 years	>12
Büyüköztürk [64]	2012	CS-1	1	10	AH + C + H+ O	OMA	300/4	CR within 4 months	n.r.
		CS-2	1	15	AH + C + H + IVIG + I	OMA	300/4	No further attacks	n.r.
von Websky [72]	2013	CR	1	n.r.	AH + C + LTRA + AB	OMA	300/4	CR = 2 days	18
Suna [73]	2009	CR	1	19	AH + C + H + IVIG + I	OMA	300/2	CR < 14 days	4.5
Stahl [43]	2014	CR	1	1	AH + C + E + H2 + TA + H + AB + LTRA + FFP + Ica	C1INH	1000 U/ twice weekly	Attack frequency decrease 5–7/month to 1.5/month	n.r.
Bayer [74]	2013	CR	1	n.r.	AH + C + E + H2 + LTRA + H + I	C1INH	n.r.	Improvement after 2 doses of C1INH	n.r.
Perez [65]	2010	CS-1	1	2.75	AH + C + I	MTX	15/1	IR = 28 days	n.r.

Dosage scheme presented as milligrams administered every *x* weeks, unless stated otherwise. The effect of treatment is presented as initial response (*IR*), complete response (*CR*), and partial response (*PR*)

*CS* case series, *CR* case report, *n.r.* not reported, *AH* antihistamine, *C* corticosteroids, *E* epinephrine, *C1-inh* C1 inhibitor concentrate, *TA* tranexamic acid, *H2* H2 antagonist, *FFP* fresh frozen plasma, *P* pantoprazole, *LTRA* leukotriene receptor antagonist, *H* hormones, *AB* antibiotics, *I* immunosuppressant, *MTX* methotrexate, *H* hydroxychloroquine, *IVIG* intravenous immunoglobulin, *Ica* icatibant, *O* others

### Ineffective Treatment Options

Ineffective treatment options were described in 21 patients in 12 articles (Table 8) [25, 31, 37, 38, 43, 48, 49, 51, 52, 60, 62, 75]. Nine of them overlap with the previously described articles since they had additionally described a successful treatment option for at least one of the subtypes of AE [25, 31, 37, 38, 43, 48, 49, 60, 62]. In three articles, only ineffective treatment options were described [51, 52, 75]. In total, ineffectiveness was recorded for TA (12 patients), C1INH and FFP (five patients each), and icatibant, MTX, and omalizumab (two patients each). In four patients, more than one therapy was recorded ineffective, in addition to conservative treatment with antihistamines, corticosteroids, and/or adrenaline. In conclusion, ineffectiveness was reported for several therapeutic options commonly used in bradykinin-mediated and mast cell-mediated AE and was reported for individual cases only, resulting in low numbers for each drug.

**Table 8:** Results of articles describing ineffective treatment

Author	Year	AE subtype	Study design	Size	Previous therapy	Ineffective therapy	Dosage
Articles describing ineffective and effective treatments							
Volans [31]	2013	ACEi-AE	CS	2	AH + C + E	TA	
Schmidt [37]	2010	ACEi-AE	CR	1	AH + C + E	C1INH	
Lipski [25]	2015	ACEi-AE	CR	1	C + E	FFP	
Dehne [38]	2007	ACEi-AE	CR	1	AH + C + E + P	FFP	
Colás [49]	2012	Idiop. (acute)	CR	1	AH + C + E + H2	C1INH	
Stahl [43]	2014	Idiop. (acute and proph.)	CR	1	AH + C + E + H2 + H + AB + LTRA	TA	
						Icatibant	
						FFP	
vd Elzen [62]	2014	AE with wheals	CS	1	AH + LTRA + I	MTX	n.r.
Vela Vizcaino [48]	2014	Idiopathic (acute + proph.)	CR	1	AH + C + E	C1INH	
Azofra [60]	2015	Idiop. (proph.)	CS	8	AH + C, or none	TA	
Articles describing ONLY ineffective treatments							
Illing [51]	2012	ACEi-AE	CR	1	AH + C + E	Icatibant	30 mg
Tran [52]	2013	Idiop. (acute)	CR	1	AH + C	FFP	n.r.
						TA	
						C1INH^a^	
Maggadottir [75]	2013	AE with wheals	CS-1	1	AH + LTRA + TCA + AB + MTX	OMA	
						MTX^b^	
	2013	AE with wheals	CS-2	1	AH + C + LTRA + IVIG + I	FFP	
						OMA	
						C1INH	

*Idiop.* idiopathic, *Proph.* prophylactic, *CS* case series, *CR* case report, *CS-x* patient number *x* in the specific case series, *n.r.* reported, *AH* antihistamine, *C* corticosteroids, *E* epinephrine, *C1-INH* C1 inhibitor concentrate, *TA* tranexamic acid, *H2* H2 antagonist, *FFP* fresh frozen plasma, *P* pantoprazole, *LTRA* leukotriene receptor antagonist, *H* hormones, *AB* antibiotics, *I* immunosuppressant, *MTX* methotrexate, *IVIG* intravenous immunoglobulin, *Ica* icatibant, *TCA* tricyclic antidepressant

^a^Icatibant effective, not included due to insufficient details

^b^IVIG effective, not included due to insufficient details

### Safety

The presence or absence of adverse effects was addressed in 25 of the 61 included articles [4–7, 19, 21, 22, 27, 42, 46, 54–60, 62–66, 70, 72, 73] (Table 9). The other 36 articles did not report information on this topic. Thus, safety information was available for 315 patients treated with either ecallantide (87 patients), icatibant (37 patients), TA (125 patients), omalizumab (34 patients), progestin (20 patients), C1INH (ten patients), or MTX (two patients).

**Table 9:** (Serious) treatment-emergent adverse events reported per treatment option

Study	Angioedema subtype	Sample size	Therapy	SAEs	No. of patients with >1 TEAE	TEAEs
Lewis [4]	ACEi-induced	58 + 18	Ecallantide	5 related SAEs (AE). One death in placebo group (respiratory compromise)	30 (51.7 %) vs. 8 (44.4 %); 13/30 related	AE (20 cases); headache and hypoesthesia (2 cases each); abdominal pain, diarrhea, hematuria, injection site pain/swelling, muscle spasms, oropharyngeal pain, oral candidiasis, pain in extremity, and pruritic rash (1 each).
Bernstein [7]	ACEi-induced	26 + 24	Ecallantide	2 (7.7 %) vs. 6 (25 %), none related	18 (75 %) vs. 17 (65.4 %); none related	n.a.
Nanda [42]	Idiopathic	1	Ecallantide	0	0	n.a.
Bas [5]	ACEi-induced	13 + 14	Icatibant	0 v.s. 1 (7 %)	1 (7 %) vs. 4 (27 %); 1/1 related	Patient-reported injection site pain; additional investigator-assessed injection site reactions in >12 (80 %)
Bova [21]	ACEi-induced	13	Icatibant	n.r.	1	Injection site pain
Bas [6]	ACEi-induced	8	Icatibant	0	8	Injection site erythema and/or itching
Crooks [27]	ACEi-induced	1	Icatibant	0	1	Injection site erythema
Lleonart [46]	Idiopathic	1	Icatibant	0	1	Injection site pain
Mansi [19]	Idiopathic	44	TA	n.r.	5	Migraine, menstrual irregularities, dyspepsia, diarrhea
Wintenberger [55]	Idiopathic	25	TA	0	11	Abdominal pain, dizziness, weakness, pain in lower limbs, migraine
Du-Thanh [58]	Idiopathic	25	TA	n.r.	1	Digestive intolerance
Firinu [56]	Idiopathic	16	TA	n.r.	Unclear^a^	Abdominal discomfort and migraine (1 case), abdominal discomfort (unclear)
Cicardi [59]	Idiopathic	15	TA	1 (myocardial infarction)	2	Laryngeal/pharyngeal dryness, self-limiting in months
Rijo Calderon [54]	AE with wheals	10	OMA	n.r.	7^a^	Drowsiness (*n* = 7), digestive, cutaneous symptoms, and weight loss (5)
Azofra [60]	Idiopathic	8	OMA	0	0	n.a.
Rijo Calderon [54]	Idiopathic	4	OMA	n.r.	7^a^	Drowsiness (*n* = 7), digestive, cutaneous symptoms, and weight loss (5)
vd Elzen [62]	AE with wheals	3	OMA	0	2	Headache in patient co-treated with cyclosporine, malaise (1 case each)
Buyukozturk [64]	Idiopathic	2	OMA	0	0	n.a.
Groffik [63]	AE with wheals	2	OMA	0	3/9^a^	Headache, blood pressure decrease, fatigue; self-limiting 3–4 days after first 3 injections
Buyukozturk [64]	AE with wheals	1	OMA	0	0	n.a.
Ghazanfar [66]	AE with wheals	1	OMA	0	0	n.a.
Sanchez-Machin [70]	AE with wheals	1	OMA	0	0	n.a.
von Websky [72]	Idiopathic	1	OMA	0	0	n.a.
Suna [73]	Idiopathic	1	OMA	0	0	n.a.
Saule [57]	Idiopathic	20	Progestin	0	17/55^a^	Weight gain (5 cases), oestrogenic deficiency (4), breakthrough bleeding (2), hyperandrogenia (2), n.r. (4)
Greve [22]	ACEi-induced	10	C1INH (B)	0	0	n.a.
Perez [65]	Idiopathic	2	MTX	1, unrelated	Unclear^a^	Hair thinning and fatigue

*Related* denote possibly, probably, or definately related to study drug, as described in the separate articles. The number of TEAEs may be higher than the number of patients reporting TEAEs since patients may have reported more than one TEAE. For RCTs, the sample size is shown as the number of treated patients + patients treated with placebo. SAE and TEAE are only shown as recorded for the treatment groups

*n.a.* not applicable since patients had reported no adverse effects or all adverse events were unrelated to the study medication

^a^TEAEs mentioned for the whole study population, which may be larger than the patients included in this review, e.g., in the case of chronic spontaneous urticaria with or without angioedema

A distinction between SAEs and less severe TEAEs was adopted from the included articles, if available. Additionally, only adverse events possibly, probably, or definitely related to treatment are shown in this review. SAEs were reported in six patients, including five AE episodes during treatment with ecallantide (6 % of those treated with ecallantide) and one myocardial infarction during treatment with TA (0.8 %). TEAEs were reported in 13 patients treated with ecallantide (15 %) and 12 treated with icatibant (32 %; all local and related to the administration method). At least 19 patients treated with TA (15 %) reported TEAE, and at least nine treated with omalizumab (26 %). However, TEAEs were presented in the total study populations including also HAE and CSU patients; therefore, the number of patients experiencing adverse effects may be higher. For progestin and MTX, TEAEs were addressed in one article each, where TEAEs were also presented in the total study population including also HAE and CSU patients. For C1INH, no TEAE was reported. In addition, one article described the use of omalizumab during two pregnancies, with no developmental abnormalities in both children [66]. In conclusion, SAEs were reported in 2 % and TEAE in 17 % of patients.

## Discussion

In this systematic review, we found several treatment options for patients with refractory AE. For acute attacks of AE, several articles described treatment with icatibant, C1INH, TA, FFP, and ecallantide. For prophylactic treatment of AE, omalizumab, TA, and C1INH were shown effective, and, with fewer included articles, also progestin and MTX. The described treatments showed good efficacy in addition to a favorable safety profile with a low number of mostly mild and self-limiting adverse effects. A limitation of the available literature was the low level of evidence for all treatment options, except ecallantide and icatibant.

In ACEi-AE, high-quality studies were performed for ecallantide, but the response rates compared to placebo were not significant. Many patients responded quickly after treatment with icatibant, C1INH, and TA, but most of the included studies were not controlled and therefore of lower quality in terms of scientific reliability. FFP has shown similar results, but since FFP also contains other substrates including prekallikrein and high-molecular-weight kininogen, it has been hypothesized to have the potential to worsen an acute attack of AE since new bradykinin can be formed [76]. Treatment of refractory ACEi-AE mostly consisted of drugs known for treatment of HAE. The rationale for this is that ACEi-AE is presumably bradykinin-mediated [2]. Icatibant had a similar time to response in ACEi-AE, as previously shown in HAE patients [77]. Ecallantide had stronger beneficial results in HAE patients [78] compared to ACEi-AE patients, partially due to a high response rate in the placebo group. Additional RCTs in HAE patients revealed time to onset of relief within 2 h for pasteurized C1INH, nanofiltered C1INH, and recombinant human C1INH (rhC1INH) [79–81]. In many of the cases included in this review, the onset of relief after C1INH was reported within 1 h of administration, and efficacy results may therefore be quite consistent with the results of C1INH treatment in acute HAE attacks. Very recently, the Canadian Agency for Drugs and Technologies in Health performed a non-systematic literature search and provided a summary of four available guidelines for urticaria and AE, which supports that icatibant, C1INH, ecallantide, and FFP may be useful in the treatment of ACEi-AE [82]. In addition to the results of these therapies in ACEi-AE, we show in the current review that these therapies, with icatibant as the most often studied, may also be effective in the treatment of acute attacks of idiopathic AE. In conclusion, in patients suffering ACEi-AE or an acute attack of idiopathic AE, ecallantide seems to have an effect in a limited number of patients, if any, whereas icatibant, C1INH, TA, and FFP often lead to symptom relief within 2 h, in addition to a good safety profile.

For AE with wheals, also known as CSU, omalizumab was the only treatment option described when conservative treatment had failed. A high success rate, good safety profile, and rapid responses were described, as was shown extensively in patients suffering CSU, which by definition includes AE with wheals [1, 10–15]. In patients suffering idiopathic AE, we show that both licensed HAE drugs and omalizumab seem to have a beneficial effect in a substantial amount of patients, even in those who are very refractory and have had many other treatments prior to the described treatment. When comparing with ACEi-AE, it appears that idiopathic AE responds even more rapidly upon treatment with icatibant, C1INH, or ecallantide. This suggests a role for both bradykinin and mast cells (histamine) in idiopathic AE with normal C1INH, although this was not the objective of the current review. Additionally, in one patient treated with C1INH and two treated with FFP, the time to response of an acute attack was reported to be 2 days [38]. In such cases, one should be aware of the natural course of an attack [1–3, 83]. Furthermore, also for this subtype, the level of evidence is low, and controlled studies remain to be performed. In conclusion, omalizumab, TA, and C1INH were effective and safe in a majority of patients in need of prophylactic treatment of refractory idiopathic AE or AE with wheals.

One needs to keep in mind that all treatment options described are currently off-label in these patient groups worldwide, except for omalizumab in AE with wheals (CSU), and that the findings should be confirmed in clinical trials. Due to the fact that most therapies described have only been registered for other indications recently, the efficacy and safety for the current subtypes of non-HAE have not been studied yet. It remains unclear which (groups of) patients derive a beneficial effect from each type of treatment. For C1INH, a beneficial effect was described even in patients who failed to respond to icatibant and/or FFP. On the contrary, in ACEi-AE and idiopathic AE, patients failed to respond to C1INH, but did respond to icatibant or TA. Similar results were seen for TA, FFP, icatibant, MTX, and omalizumab, indicating the presence of non-responders for each type of treatment in almost each subtype of AE and also indicating that switching treatment options can lead to satisfactory results in some individuals even when both target a similar pathophysiological mechanism.

We opted for a broad overview of the level of evidence of treatment options when performing this systematic review. This was deemed appropriate with regard to the research question and the therapeutic problems physicians face in daily practice. The results may be an overestimation since case reports generally represent one or few patients with positive effects of treatment, and only few cases without response are available possibly due to underreporting. Due to the use of different outcome measures, such as percentage of patients with response or the time to response, it was difficult to compare the results of the studies. Additionally, we found there to be a low level of prior research evidence. Fortunately, in the last couple of years, more extensive research has been published, allowing for the inclusion of several RCTs in this review. Still, our results illustrate the need for further research in these patient groups, including prospective cohort studies and controlled studies. The lack of available guidelines underlines this further. Not included in this review but worthy of mention is the fact that it is known that AE is known to have a detrimental effect on quality of life (QoL) [84]. While the impact on the QoL was not a part of this review, it is striking that this aspect was not addressed in many of the included studies. Disease-specific questionnaires have been developed for AE patients, both with regard to disease activity and QoL [1, 84–86], and we consider QoL an important additional outcome measure both in acute attacks and prophylactic setting studies.

A minority of articles included information with respect to adverse effects of treatment. When reported, only a few patients experienced adverse effects. These were generally mild and self-limiting, and most were known side effects [15, 76, 87–91]. New TEAEs were oropharyngeal discomfort (reported for TA), weight loss (omalizumab), and hypoesthesia, hematuria, muscle spasms, oral candidiasis, and pain in extremity (ecallantide; TEAEs may be unrelated). Notably, for icatibant, only injection-related TEAEs occurred.

In conclusion, for patients suffering angioedema refractory to conservative treatment, several additional treatment options are available with rapid time to response, high response rates, and limited side effects. However, these therapies are off-label, and there is a need for additional studies to provide a high level of scientific evidence. Treatment options differ per subtype of AE. Most promising treatments for acute attacks (ACEi-AE and idiopathic AE) consist of icatibant, C1INH, and FFP, with response often within 2 h and with limited side effects. For prophylactic treatment (idiopathic AE and AE with wheals), the most promising options are omalizumab, TA, and C1INH, with efficacy in a majority of patients, together with limited side effects.

## Abbreviations

ACEi-AE: Angiotensin-converting enzyme inhibitor-induced angioedema
AE: Angioedema
C1INH: C1 esterase inhibitor
CSU: Chronic spontaneous urticaria
FFP: Fresh frozen plasma
fXII: Coagulation factor 12
HAE: Hereditary angioedema
RoB: Risk of bias
MTX: Methotrexate
QoL: Quality of life
RCT: Randomized controlled trial
SAE: Serious adverse event
TA: Tranexamic acid
TEAE: Treatment-emergent adverse event
